# Neural Correlates of Fear in the Periaqueductal Gray

**DOI:** 10.1523/JNEUROSCI.1100-16.2016

**Published:** 2016-12-14

**Authors:** Thomas C. Watson, Nadia L. Cerminara, Bridget M. Lumb, Richard Apps

**Affiliations:** ^1^School of Physiology, Pharmacology and Neuroscience, Biomedical Sciences Building, University of Bristol, University Walk, Bristol BS8 1TD, United Kingdom, and; ^2^Neuroscience Paris Seine, Cerebellum, Navigation, and Memory Team, Sorbonne Universities, Université Pierre et Marie Curie, University of Paris 06 Unité Mixte de Recherche Scientifique 8246, INSERM Unité Mixte de Recherche Scientifique 1130, and Centre National de la Recherche Scientifique Unité Mixte de Recherche 8246, F-75005 Paris, France

**Keywords:** EMG, extinction, fear, fear conditioning, freezing, periaqueductal gray

## Abstract

The dorsal and ventral periaqueductal gray (dPAG and vPAG, respectively) are embedded in distinct survival networks that coordinate, respectively, innate and conditioned fear-evoked freezing. However, the information encoded by the PAG during these survival behaviors is poorly understood. Recordings in the dPAG and vPAG in rats revealed differences in neuronal activity associated with the two behaviors. During innate fear, neuronal responses were significantly greater in the dPAG compared with the vPAG. After associative fear conditioning and during early extinction (EE), when freezing was maximal, a field potential was evoked in the PAG by the auditory fear conditioned stimulus (CS). With repeated presentations of the unreinforced CS, animals displayed progressively less freezing accompanied by a reduction in event-related field potential amplitude. During EE, the majority of dPAG and vPAG units increased their firing frequency, but spike-triggered averaging showed that only ventral activity during the presentation of the CS was significantly coupled to EMG-related freezing behavior. This PAG–EMG coupling was only present for the onset of freezing activity during the CS in EE. During late extinction, a subpopulation of units in the dPAG and vPAG continued to show CS-evoked responses; that is, they were extinction resistant. Overall, these findings support roles for the dPAG in innate and conditioned fear and for the vPAG in initiating but not maintaining the drive to muscles to generate conditioned freezing. The existence of extinction-susceptible and extinction-resistant cells also suggests that the PAG plays a role in encoding fear memories.

**SIGNIFICANCE STATEMENT** The periaqueductal gray (PAG) orchestrates survival behaviors, with the dorsal (dPAG) and ventral (vPAG) PAG concerned respectively with innate and learnt fear responses. We recorded neural activity from dPAG and vPAG in rats during the expression of innate fear and extinction of learned freezing. Cells in dPAG responded more robustly during innate fear, but dPAG and vPAG both encoded the time of the conditioned stimulus during early extinction and displayed extinction sensitive and resistant characteristics. Only vPAG discharge was correlated with muscle activity, but this was limited to the onset of conditioned freezing. The data suggest that the roles of dPAG and vPAG in fear behavior are more complex than previously thought, including a potential role in fear memory.

## Introduction

The execution of defensive responses to fearful, often life-threatening, stimuli is fundamental to survival (Fanselow, 1994). Such behavioral responses are complex and encompass active (engagement) and passive (disengagement) strategies including fight/flight and freezing respectively, which can be innate or learned (conditioned; Zhang et al., 1990; Bandler et al., 2000; Wang et al., 2015).

The neural network components for innate and conditioned fear include, but are not limited to, the prefrontal cortex, hippocampus, amygdala, hypothalamus, and different subdivisions of the periaqueductal gray (PAG) (Tovote et al., 2015; Furlong et al., 2016). It is generally thought that the PAG is downstream of the amygdala and prefrontal cortex, generating the appropriate defensive response to fearful or threatening events (Tovote et al., 2016). The networks that coordinate innate behaviors include dorsal regions of the PAG (dorsal PAG, dPAG; including dorsal, dorsolateral, and lateral columns; Bandler and Depaulis, 1988; Fanselow et al., 1991; Walker and Davis, 1997; De Oca et al., 1998; Vianna et al., 2001a), whereas those that orchestrate learned behaviors include the ventral PAG (vPAG; notably its ventrolateral column; Bandler and Shipley, 1994; Bandler et al., 2000; Vianna et al., 2001b; Helmstetter et al., 2008). Depending on context (e.g., proximity of threat), both innate and learned defense behaviors include freezing (Bowen et al., 2013; Koutsikou et al., 2014).

Neuronal recordings in awake animals have provided important insights into the roles of high-order components of survival networks in defense behaviors (e.g., the amygdala; Muramoto et al., 1993; Quirk et al., 1995; Herry et al., 2008; Duvarci et al., 2011). However, only limited information has been derived from equivalent neuronal recordings in the PAG during freezing behavior evoked by innate or conditioned stimuli.

To date, studies have focused on PAG activity in relation to expectancy of an unconditioned stimulus (Johansen et al., 2010) or retrieval of a conditioned response (Halladay and Blair, 2015; Tovote et al., 2016). In addition, the extent of neural coupling with motor behavior and the differences between dPAG and vPAG in relation to extinction of conditioned fear are unknown. This is an important gap in understanding because extinction is a key feature of associative learning, enabling an animal to adapt to a changing environment (Bouton, 2004). Deficits in the normal extinction learning process are also thought to underlie anxiety disorders (Milad et al., 2008).

In light of this lack of knowledge, we combined neuronal recordings (field potential and single unit activity) in the dPAG and vPAG before, during, and after the behavioral changes evoked by exposure to innate or conditioned fear stimuli. Simultaneous monitoring of neck muscle EMG also allowed us to relate the timing of changes in motor output associated with freezing behavior to the patterns of neural activity (Steenland and Zhuo, 2009). This allowed us to interrogate the roles of dPAG and vPAG in relation to motor activity associated with innate fear and extinction of a conditioned fear stimulus.

## Materials and Methods

### 

#### 

##### Implant procedures.

All animal procedures were performed in accordance with the UK Animals (Scientific Procedures) Act of 1986 and was approved by the University of Bristol Animal Welfare and Ethical Review Body. A total of 17 adult male Wistar rats (300–400 g; Charles River Laboratories and Harlan Laboratories) were used in this study. They were housed under normal environmental conditions (20°C and 45–65% humidity) on a 12 h dark/light cycle and provided with food and water *ad libitum*.

Rats were anesthetized either with a mixture of isoflurane and O_2_ (*n* = 10 rats) or by intraperitoneal injection with ketamine and medetomidine (*n* = 7 rats, 5 mg/100 g of Vetalar, Boehringer Ingelheim; 30 μg/100 g of Domitor, Pfizer). Each animal was mounted in a stereotaxic apparatus with atraumatic ear bars and surgery was performed under aseptic conditions. Depth of anesthesia was checked regularly by testing for corneal and paw withdrawal reflexes and the level of gaseous anesthetic was adjusted or supplementary doses of ketamine given as required.

A midline scalp incision was made and a craniotomy performed to gain access to the PAG (7.5 mm caudal from bregma, 1 mm lateral from midline). An in-house-built miniature microdrive was attached to the skull with screws and dental acrylic cement. The microdrive contained 1–4 tetrodes for LFP and single unit recordings (tungsten, 12.5 μm inner diameter, impedance 100–300 kΩ after gold plating; California Fine Wire). The tetrodes were stereotaxically lowered through the craniotomy to a position just dorsal to the PAG (∼4 mm below the brain surface). A pair of flexible, stainless steel insulated wires (Cooner) were also sutured into neck muscle to record EMG as a marker of freezing behavior (Steenland and Zhuo, 2009). These leads were fed subcutaneously to the microdrive and the skin incision closed in layers.

##### Behavioral and electrophysiological recording procedures.

Before surgery, animals were habituated to handling for at least 2 d. One week after surgery, rats were handled for at least another 2 d before daily recording sessions commenced. In these sessions, the position of the tetrodes was adjusted to obtain single unit activity within either the dPAG or vPAG (4.0–4.5 or 4.6–5.6 mm from the brain surface, respectively). Once single units were localized, the electrode was kept in the same position throughout behavioral testing (i.e., the same recording position was maintained for experimental days 0–4).

Fear conditioning and extinction testing occurred in different contexts (contexts A and B). The Skinner box (Med Associates) was dimly lit and located within a soundproofed room. The walls, ceiling, and floor were cleaned with 70% ethanol after each session. Context A had a clear Perspex back wall, ceiling, and front door with aluminum sidewalls and a metal grid floor. For context B, the inner structure of the chamber was altered through the addition of a white plastic floor, striped wall, and a tissue impregnated with vanilla essence placed under the flooring. For habituation and fear conditioning (days 0–2), the animals were placed in context A, whereas during extinction testing, they were placed in context B (day 3).

On experimental day 0, the rats were habituated to the conditioning chamber for 5 min. On experimental day 1, after a 5 min acclimatization period to context A, the rats received an auditory habituation session consisting of 7 auditory tones (each tone 10 s, 1 kHz, 80 dB). Experimental day 2 consisted of a 5 min acclimatization period to context A followed by the fear conditioning session, when the rats received 7 conditioned stimulus (CS) presentations of the same auditory tone as day 1, but with each tone coterminating with a foot-shock unconditioned stimulus (0.75 mA, 1 s). On experimental day 3, after a 5 min acclimatization period, the rats received an extinction session consisting of CS-only trials in context B (each tone 10 s, 1 kHz, 80 dB). The CS-only trials were in blocks of 7 tones (time interval between tones in each block was 30 s) and 7 blocks of trials were presented during the session (time interval between each block was 2 min). In keeping with a previous study (Burgos-Robles et al., 2009) CS-only block 1 was defined as early extinction (EE; equivalent to CS retrieval), whereas CS-only block 7 was defined as late extinction (LE). At the end of the CS-only session, rats were returned to their home cages. After 24 h (experimental day 4), a piece of filter paper impregnated with cat odor was placed in the top of their home cages to test innate fear responses (exposure duration ∼3 min; Koutsikou et al., 2014).

Electrophysiological data were obtained during experimental days 1, 3, and 4 (recordings were not possible on experimental day 2 because of stimulus and movement artifacts during the acquisition phase of fear conditioning). Signals were captured using a Lynx 8 acquisition system (Neuralynx) and CED Power1401 (Cambridge Electronic Design). LFP and EMG signals were sampled at 5 kHz and band-pass filtered between 0.1 and 600 Hz. For the majority of recordings, neural signals were sampled at 20 kHz and band-pass filtered between 600 Hz and 6 kHz. Single unit and LFP recordings were referenced to a skull screw positioned over the cerebellum, which served as the indifferent electrode. EMG signals were recorded in a bipolar configuration with wires positioned on either side of the neck (cf. Steenland and Zhuo, 2009).

Throughout all sessions, behavior was monitored by video and scored offline. Time spent freezing (defined as the cessation of all movements except those associated with respiration and eye movements; Blanchard and Blanchard, 1969) was assessed using a combination of video and neck EMG recording (smoothed using a 25 ms time constant and rectified; Steenland and Zhuo, 2009).

##### Data analysis.

All data were tested for a Gaussian distribution using the D'Agostino and Pearson omnibus normality test and parametric or nonparametric statistical analysis was performed as appropriate. Data were analyzed offline with Spike2 software (Cambridge Electronic Design), MATLAB (The MathWorks), and Neuroexplorer (Nex Technologies). For extinction sessions (CS-only trials), freezing was measured as the percentage of time immobile during the 7 × 10 s duration of the CS and the 7 × 30 s duration of the intertrial interval. A repeated-measures ANOVA with Tukey's *post hoc* test was used to compare freezing rates at different time points throughout the protocol (from baseline, a period of 3 min when animals were in the conditioning box before playing of the CS, through to CS block 7). For the innate fear session (cat odor presentation), freezing was measured as the percentage time spent immobile during the 3 min exposure to cat odor.

We also recorded LFP from seven animals during tone habituation (the presentation of the same tone used for conditioning, but presented seven times before conditioning). To test changes in tone evoked field potentials during habituation, we constructed field potential averages from tones 1–3 and 5–7. The amplitude of these field potentials was then compared using a paired *t* test.

For field potential analysis during extinction training, average waveforms were constructed using tone onset as the trigger (7 tones per average, *n* = 10 rats; 4 animals were excluded from analysis due to poor signal-to-noise recording conditions). The amplitude of responses evoked during EE and LE were compared using a paired *t* test. Pearson correlation was also used to assess the relationship between event-related field potential amplitude and level of freezing behavior.

Spike sorting was performed using a clustering algorithm based on template matching and principal component analysis. Single units were subdivided into two groups according to recording depth: (1) dorsal units (depth 4.0–4.5 mm from the surface of the brain) or (2) ventral units (4.6–5.6 mm from the surface of the brain).

To detect temporal changes in single unit activity, we divided the duration of the CS tone into 10 bins, each 1 s in duration. A *z*-score for each of these bins was calculated relative to 10 pre-tone bins of equal duration (obtained in the same session). Units were classified as showing significant increases or decreases in response to the CS if any bins within the tone exceeded a *z*-score of +1.96 or a *z*-score of ≤1.96, respectively (*p* < 0.05, two tailed test). To detect changes in firing rate, we compared peak *z* values (1 value per animal) during EE and LE (independently of the time bin in which the peak in firing frequency occurred) using Mann–Whitney *U* tests. Response latencies were computed using peristimulus time histogram (PSTH) plots with 10 ms time bins and calculated as the time interval from the onset of the CS to the first bin exceeding 95% confidence level.

Group perievent time histograms were generated by averaging *z*-scores of unit activity before and after tones or exposure to the cat odor. Mann–Whitney *U* tests were used to examine differences in unit firing rate. A χ^2^ test was used to detect differences in the proportion of tone-responsive units in EE versus LE (see above) and also in relation to dorsal versus ventral recording site position. Type 1 neurons (see Results) were the most common cell type with sufficient sample size to carry out further analysis. For type 1 cells, Spearman's correlation was used to assess the relationship between PAG firing rate and time spent freezing and spike-triggered averages (STAs) were constructed of EMG in relation to their activity.

The STA analysis was obtained for a sample of cases (*n* = 9 rats) in relation to the following: (1) EE during the 10 s of CS presentation, (2) EE 10–20 s after the CS, and (3) EE during quiet immobility (based on a 200 s sample of recording preconditioning). Animals were selected for this stage of analysis based upon the quality of their EMG signals. We excluded all animals in which large amplitude artifacts contaminated the recordings. For all three conditions (during the CS, after the CS, and during quiet immobility), the rectified EMG was triggered from spikes recorded from type 1 units located in either dPAG or vPAG. Due to variations in the EMG amplitude, the data were *z*-score normalized before averaging across animals. Latency to peak EMG response was calculated from individual STA plots. Mann–Whitney tests were used to compare amplitudes and latencies of the averages. All values are shown as mean ± SEM and *p* < 0.05 was selected as the criterion for statistical significance.

##### Histology.

At the end of every experiment, animals were deeply anesthetized (Euthatal, 200 mg ml^−1^, Merial Animal Health) and electrolytic lesions performed to mark the position of tetrode tips. The animals were then perfused (4% paraformaldehyde in 0.1 m phosphate buffer) and the brains extracted. After postfixation, the brains were cryoprotected in 30% sucrose solution and coronally sectioned at 60 μm.

## Results

### Freezing behavior: conditioned and innate fear

**Figure 1.**
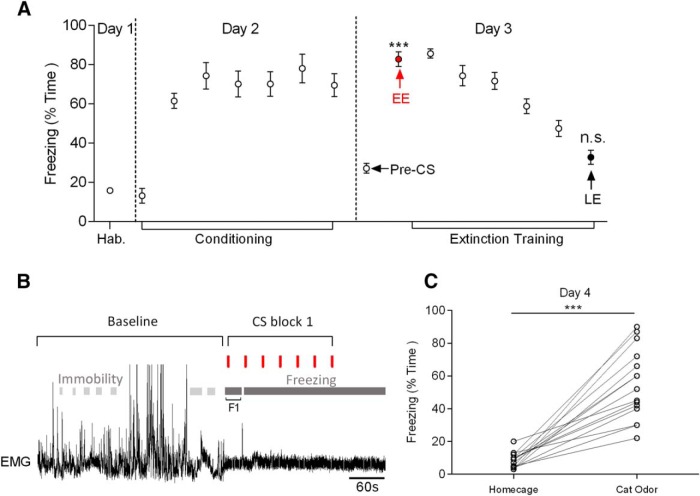
Freezing levels during conditioned and innate fear. ***A***, Percentage of time freezing during tone habituation (day 1), auditory fear conditioning (day 2), and pre-CS baseline and auditory conditioned fear extinction (day 3). Color-coded data points (mean ± SEM) indicate times when single unit and LFP data were analyzed. For EE, *n* = 17 rats (red filled circle); for LE, *n* = 14 rats (black filled circle). ns, *p* > 0.05; ***p < 0.001, ANOVA with Tukey's post test against baseline. ***B***, Example of immobility detection before (baseline) and freezing detection during exposure to an auditory CS (CS block 1, onset of each tone is indicated by red lines). F1 is the initial period of maintained freezing after presentation of the first CS. In this example, this is interrupted by a brief period of movement before a subsequent extended period of further freezing. ***C***, Freezing time in the home cage after extinction training and during exposure to cat odor (day 4; *n* = 14 rats; ***p < 0.001, paired *t* test).

Figure 1*A* shows grouped data of the duration of time that rats displayed freezing behavior during auditory conditioned fear acquisition and extinction (experimental days 2 and 3, *n* = 17 rats) and also during subsequent exposure to cat odor to induce innate fear (experimental day 4, *n* = 14 of the same rats; Fig. 1*C*). Consistent with previous studies (Duvarci et al., 2011; Koutsikou et al., 2014), exposure to a CS tone previously associated with an aversive foot shock produced a statistically significant increase in freezing during EE (CS blocks 1–3; ANOVA with Tukey's *post hoc* test between baseline and CS blocks 1, 2, or 3, *p* < 0.0001, *F*_(8,90)_ = 40.6, *n* = 17) compared with preconditioning baseline. For example, during CS block 1, animals spent 82.8 ± 4% of their time freezing compared with 27.1 ± 3% of their time during baseline trials (Fig. 1*A*). The duration of maintained freezing initiated upon presentation of the first CS (an example is shown in Fig. 1*B*) varied considerably between animals (ranging from 18 to 470 s). However, the duration of freezing always substantially outlasted the initial 10 s tone (mean first freeze duration = 94.5 ± 41.9 s). In keeping with previous investigations, the proportion of time that the animals displayed freezing gradually decreased with subsequent blocks of CS-only trials and were similar to baseline levels by CS block 7 (LE, 32.8 ± 4% time spent freezing, ANOVA with Tukey's *post hoc* test vs baseline, *p* > 0.05, *n* = 14; 3 rats did not undergo the full sequence of extinction training).

On experimental day 4, the same rats were exposed to cat odor (see Materials and Methods) and in agreement with previous studies (Koutsikou et al., 2014), they displayed a statistically significant increase in innate freezing behavior (on average, they spent 54.9 ± 6% of their time freezing, ANOVA with Tukey's *post hoc* test, *p* < 0.05 vs baseline, *n* = 14; Fig. 1*C*).

### dPAG and vPAG recordings: innate fear

**Figure 2.**
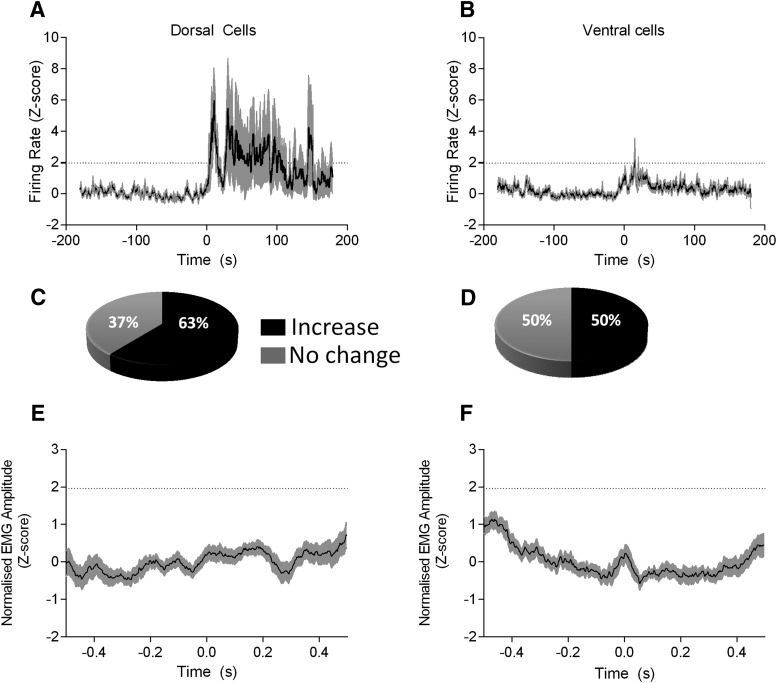
PAG cell responses to cat odor. ***A***, Grouped *z*-score plots of dPAG unit activity during exposure to cat odor (onset at time 0, average of *n* = 23 units). ***B***, Same as ***A*** but grouped responses of vPAG units (average of *n* = 24 units). Black line represents mean; shaded area indicates SEM, 1 s bins. Dotted horizontal lines indicate *p* = 0.05. Pie charts (***C***, dorsal; ***D***, ventral units) to indicate proportions of response patterns to the stimulus. ***E***, ***F***, Spike-triggered EMG averages using spike activity of dPAG and vPAG units, respectively. EMG data were rectified, smoothed (0.025 s), and expressed as a *z*-score. Shading indicates SEM. Dotted horizontal lines indicate confidence level at *p* = 0.05.

The activity of single units in the dPAG and vPAG was examined during innate fear elicited by exposure to cat odor (Fig. 2). Grouped analysis revealed that dPAG units displayed a statistically significant greater increase in firing rate than vPAG units during initial exposure to the innate fear-inducing stimulus (i.e., during the first 20 s after cat odor exposure; *z*-score 2.7 ± 0.4 vs 0.9 ± 0.1 *z*-score, respectively, *p* < 0.0001, Mann–Whitney *U* value = 68, *n* = 14 rats; Fig. 2*A*,*B*). Furthermore, individual unit analysis revealed that 63% (14/23) of dPAG units and 50% (12/24) of vPAG cells displayed significant increases in firing rate during the first 20 s after exposure to the cat odor (Fig. 2*C*,*D*). There were no significant reductions in firing rate in response to cat odor. To investigate the relationship between PAG firing and muscle activity, we constructed STAs of neck EMG after exposure to cat odor. No statistically significant relationship was found for STAs constructed in relation to either dPAG or vPAG unit activity (Fig. 2*E*,*F*).

### dPAG and vPAG recordings: conditioned fear

**Figure 3.**
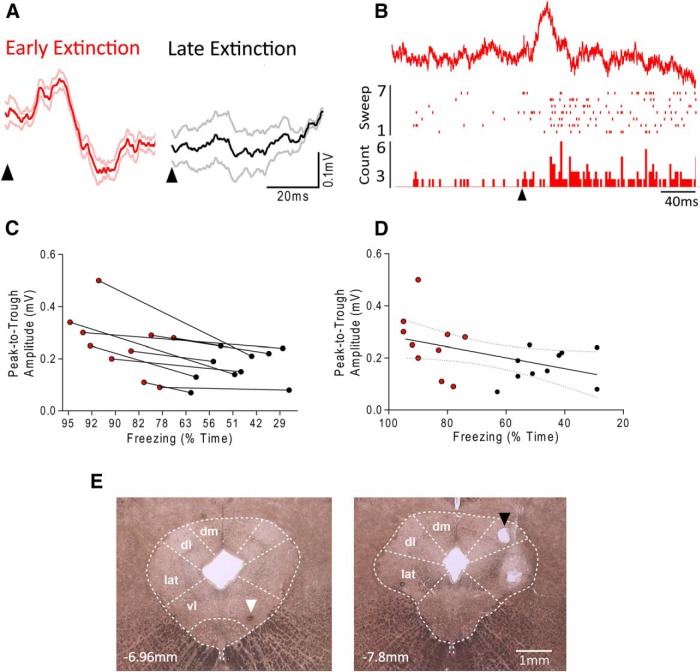
Tone-evoked PAG local field potentials during conditioned fear behavior. ***A***, Example of event-related field potential recorded in the PAG obtained from one animal (average of seven trials triggered relative to tone onset, arrowheads). Event related field response recorded from same recording site is shown during EE and LE. Solid lines indicate the mean; light red and gray lines, SEM. ***B***, Simultaneously recorded event-related field potential (average of seven trials; top trace) and single unit (raster and PSTH) in dPAG during EE. Tone onset is indicated by arrowhead. PSTH bins are 2 ms. ***C***, Relationship between amplitude of tone-evoked field and percentage of recording session time displaying freezing (*n* = 10 animals, 2 data points per animal; EE, red and LE, black). ***D***, Correlation plot of evoked field amplitude as a function of time spent freezing. Solid line indicates linear regression and dotted lines represent 95% confidence intervals. ***E***, Coronal sections of the PAG showing examples of electrolytic lesions performed at the end of the experiments to mark recording sites. The photomicrograph to the left shows a case with a lesion in vPAG (white arrowhead) and the photomicrograph to the right shows a different case with a lesion in dPAG (black arrowhead). Approximate position of section relative to bregma is indicated. dm, Dorsomedial; dl, dorsolateral; lat, lateral; vl, ventrolateral.

For studying neural activity during conditioned fear, in initial experiments, we recorded field potentials in PAG evoked by the CS tone (*n* = 10 rats; Fig. 3*A*). Histological verification of example recording positions in dPAG or vPAG are shown in Figure 3*E*. Because the event related responses were similar for both dorsal and ventral recording sites, the data were pooled.

On average, the onset and peak latencies of the event-related field during EE were 9 ± 1 ms and 20 ± 2 ms, respectively (Fig. 3*A*,*B*). The average duration of the event-related field was 37 ± 10 ms. In cases in which the event-related field was recorded simultaneously with single unit activity, the time course of the field coincided with an increase in neuronal firing frequency (Fig. 3*B*). Field potentials mainly reflect synaptic activity, so if the unit activity and field potential activity are related, then it would be expected that evoked spikes would occur later than the onset of the field. In the example illustrated in Figure 3*B*, the event-related field occurs from ∼8–45 ms, whereas the onset latency of single unit firing occurs at ∼35 ms.

In every available case (*n* = 10), the event-related field was reduced in amplitude in LE (Fig. 3*A*,*C*). The mean peak-to-trough amplitude of the response was 0.26 ± 0.04 mV during EE, but only 0.16 ± 0.02 mV during LE, representing an ∼40% reduction in size (*p* = 0.01, paired *t* test, *n* = 10 rats). Correlation analysis revealed a statistically significant positive relationship between the amount of time spent freezing and the amplitude of the tone evoked field potential (*r* = 0.45, *p* = 0.04; Fig. 3*D*). In seven of the same animals, we also recorded event-related field potentials during tone habituation before conditioning. In contrast to responses evoked by the same tone during extinction training, there was no significant difference in field potential amplitude during habituation (the average peak-to-trough amplitude during tones 1–3 was 0. 17 ± 0.04 mV and 0.18 ± 0.04 mV during tones 5–7; *p* = 0.56; paired *t* test).

Having established that the CS could evoke substantial field potentials in PAG that were reduced by extinction training, our next step was to investigate the activity patterns of single units. Unit recordings were obtained from the PAG in 14 rats during EE and in 12 of the same rats during LE. Offline clustering (see Materials and Methods) of unit activity yielded a total sample of 73 units during EE (41 located within dPAG and 32 located within vPAG, *n* = 14 rats) and 50 units during LE (28 located within dPAG and 22 located within vPAG, *n* = 12 rats). Two animals did not undergo the full sequence of extinction training, leading to a smaller sample size in LE (recordings from these two animals contributed three dorsal and five ventral cells, respectively, to the total unit count during EE). Loss of single unit recording from individual cells during the full sequence of extinction training was 20% across all available animals.

**Figure 4.**
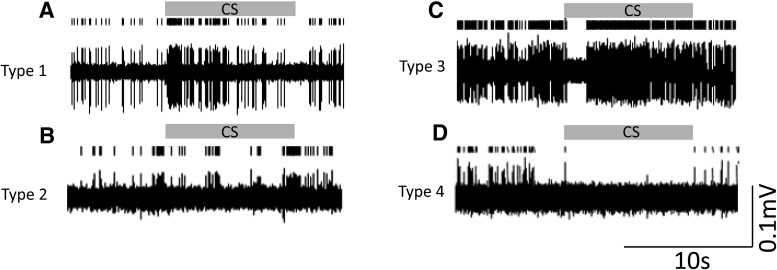
Different types of response of units in PAG. Shown are examples of the different patterns of response to the CS during EE (tone block 1, gray horizontal bar CS, conditioned stimulus). ***A***, Type 1, increased firing. ***B***, Type 2, no significant change in firing. ***C***, Type 3, biphasic response. ***D***, Type 4, decreased firing. Examples in ***A*** and ***C*** were units located in the dPAG and examples in ***B*** and ***D*** were units located in the vPAG.

Figure 4 shows examples of unit activity during presentation of the CS during EE. The sample of PAG units could be classified into four types. The most common type of response was an increase in firing rate (type 1, representing 67% of the total population; Fig. 4*A*), whereas no significant change in firing (type 2; Fig. 4*B*) or a biphasic pattern of response (type 3; Fig. 4*C*) each represented 14.5% of the population sample. The fourth class were units that displayed a reduction in firing rate (type 4, representing only 4% of the population sample recorded in EE; Fig. 4*D*).

**Figure 5.**
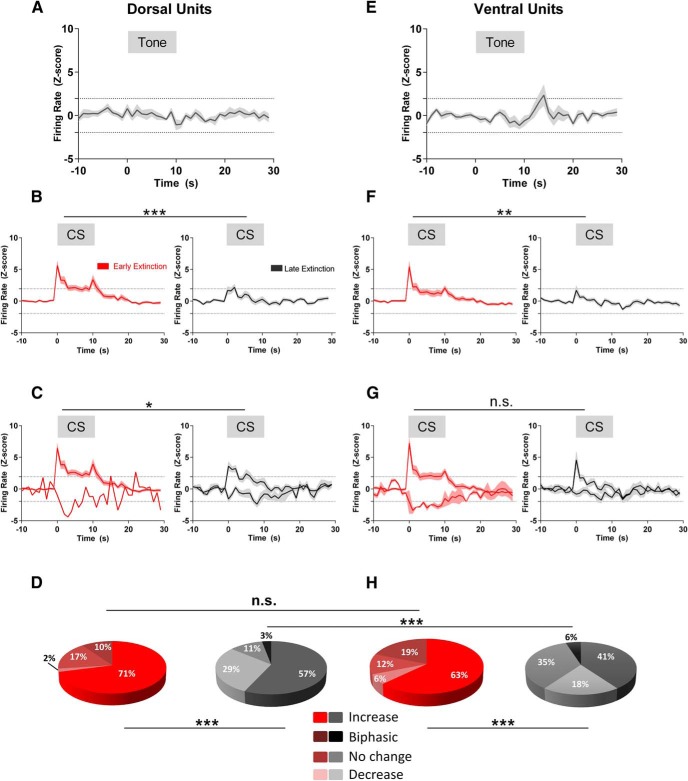
Changes in firing pattern during extinction training. ***A***, ***E***, Firing patterns of cells recorded in dPAG and vPAG during presentation of an initially neutral auditory tone (gray horizontal bar, *n* = 10 dorsal and *n* = 10 ventral cells, *n* = 6 rats). ***B***, ***F***, Pooled firing rate plots of all dorsal (left, *n* = 41 cells, 14 rats; right, *n* = 28 cells, 12 rats) and ventral (left, *n* = 32 cells, 14 rats; right, *n* = 22 cells, 12 rats) cells during EE (red) and LE (black), respectively. ***C***, ***G***, Average of a subset of dorsal (left, *n* = 30 cells, 14 rats; right, 24 cells, 12 rats) and ventral (left, *n* = 22 cells, 14 rats; right, *n* = 13 cells, 12 rats) PAG cells with statistically significant increases or decreases (*p* ≤ 0.05) in activity during the CS (in ***B***, ***C***, ***F***, and ***G*** shown as gray horizontal bar). ***D***, ***H***, Pie charts showing proportion of dorsal and ventral cells with different response types during early (red) and late (black) extinction, respectively. For *z*-score plots, average is represented by solid lines and shading indicates the SEM. Dotted horizontal lines indicate confidence level at *p* = 0.05. Bins are 1 s. ***p <0.001; **p < 0.01; *p < 0.05, n.s., nonsignificant, Mann–Whitney test between peak responses. For pie charts, ***p < 0.001. n.s., nonsignificant, χ^2^ test.

The different types of activity were present for units located in both dPAG and vPAG (see below). To explore the activity in more detail, firing rate averages were obtained at three different time points in relation to presentation of the auditory tone: (1) in the preconditioning habituation phase (*n* = 6 rats), (2) postconditioning during EE (CS block 1, *n* = 14 rats), and (3) postconditioning during LE (CS block 7, *n* = 12 rats). Figure 5 displays the data for all units located in dPAG or vPAG grouped separately to show population averages for the different regions of PAG as a function of extinction training.

A total of 10 dPAG units (mean baseline firing rate 2.3 ± 0.3 Hz) were recorded during habituation to the tone and none displayed a statistically significant response (Fig. 5*A*). In contrast, during EE, grouped analysis revealed that units recorded from dPAG displayed a statistically significant increase in firing rate around the onset and offset of the CS (onset latency 35.8 ± 6.7 ms; *n* = 41 units; Fig. 5*B*, left plot). This phasic increase in firing rate was absent in LE (Fig. 5*B*, right plot) and the difference in peak firing rate between EE and LE was statistically significant (*z*-score 5.6 ± 0.7 vs 1.7 ± 0.6, *p* = 0.0005, Mann–Whitney *U* value = 389, *n* = 41 cells, 14 rats during EE and *n* = 28 cells, 12 rats during LE, respectively).

When the analysis was performed on the firing patterns of individual units located in dPAG, a statistically significant increase in firing rate throughout presentation of the CS in EE was found in 71% of the sample (29/41 units; Fig. 5*C*, left); of the remainder, 27% (11/41 of units) displayed either a biphasic or no significant change in firing rate, whereas only 2% (1/41) showed a significant reduction (Fig. 5*C*, left, 5*D*, red pie chart). By comparison, during LE, 57% of dPAG units (16/28) significantly increased their firing rate, 29% (8/28 units) displayed a significant decrease (Fig. 5*C*, right), and the remainder (14%, 4/28 units) showed either a biphasic response or no change in activity during presentation of the CS (Fig. 5*D*, gray pie chart). Therefore, dPAG units were 29 times more likely to display an increase than a decrease in firing rate during EE. This ratio reduced to a twofold difference in LE. For those dPAG cells that displayed a statistically significant increase in activity during EE, the peak firing rate was significantly higher during the CS in EE compared with the sample of dPAG cells that displayed a significant increase in LE (*z*-score 6.5 ± 0.7 versus 3.6 ± 0.7; *p* = 0.04, Mann–Whitney *U* value = 239; *n* = 29 units and *n* = 16 units, respectively, obtained from 14 and 12 rats; Fig. 5*C*).

A total of 10 vPAG units (mean baseline firing rate 2.8 ± 0.6 Hz) were recorded during habituation to the auditory tone and none displayed a statistically significant response (Fig. 5*E*). During EE, grouped analysis revealed that vPAG units (like dPAG units) displayed a statistically significant increase in firing rate during CS presentation, particularly at the onset of the tone (onset latency 34.2 ± 5.3 ms; *n* = 32 units; Fig. 5*F*, left) and, as a population, this effect was absent during LE (*n* = 22 units; Fig. 5*F*, right). The overall reduction in mean firing rate between EE and LE was statistically significant (*z*-score 5.4 ± 0.9 vs 1.7 ± 0.7, *p* = 0.028, Mann–Whitney *U* value = 219; *n* = 32 cells and *n* = 22 cells, respectively, obtained from 14 and 12 rats). The peak in activity at the onset of the CS suggests that at least some vPAG cells are not encoding the CS–US interstimulus interval (otherwise, there would be a sustained change in firing during CS tone delivery and/or increased activity at offset, when the US would be predicted to occur). However, to test this possibility would require further experiments in which the pattern of response of individual cells was recorded in relation to a range of different interstimulus intervals.

Individual unit analysis revealed that, during EE, the majority of vPAG units (63%, 20/32) showed a statistically significant increase in firing rate during CS presentation, whereas 31% (10/32) displayed either a biphasic response or no significant change and 6% (2/32) showed a significant decrease in firing rate (Fig. 5*H*, red pie chart). During LE, the proportions were 41% (9/22 units) that displayed an increase; 18% (4/22) displayed a reduction, and 41% (9/22) displayed either a biphasic response or no change in firing rate (Fig. 5*H*, gray pie chart). Therefore, vPAG units were 10 times more likely to display an increase than a decrease in firing rate during EE. Like dPAG units, this ratio reduced to an approximately twofold difference in LE. However, in contrast to dPAG activity, when the vPAG units with a statistically significant increase in firing rate during CS presentation in EE were compared with the sample of vPAG cells that displayed a significant increase in LE, the increase was similar in magnitude (*z*-score 7.2 ± 1.0 versus 4.6 ± 1.3; ns, *p* = 0.17, Mann–Whitney *U* value = 83; *n* = 20 units and *n* = 9 units, respectively, obtained from 14 and 12 rats; Fig. 5*G*). In other words, those vPAG units that did respond with an increase in activity during LE did so robustly.

When the proportion of units displaying the 4 different types of response (type 1, an increase; type 2, no change in activity; type 3, a biphasic response; or type 4, a decrease) were compared, there was a significant difference in EE versus LE for both dPAG and vPAG units (for dorsal cells, χ^2^ = 30.1, df = 3, *p* < 0.001; for ventral cells, χ^2^ = 32.1, df = 3, *p* < 0.001; Fig. 5*D*,*H*). When a comparison was made between dPAG and vPAG units during EE, there was no significant difference in the proportion of the different types of response (χ^2^ = 6.1, df = 3, *p* = 0.11; Fig. 5*D*,*H*, red pie charts). In contrast, during LE, dPAG and vPAG units displayed a statistically significant difference in the prevalence of firing patterns: a greater proportion of dorsally located cells were type 1 units compared with ventrally located units (χ^2^ = 17.7, df = 3, *p* = 0.0005; Fig. 5*D*,*H*, gray pie charts).

### Relationship between unit activity and freezing behavior/EMG during extinction

**Figure 6.**
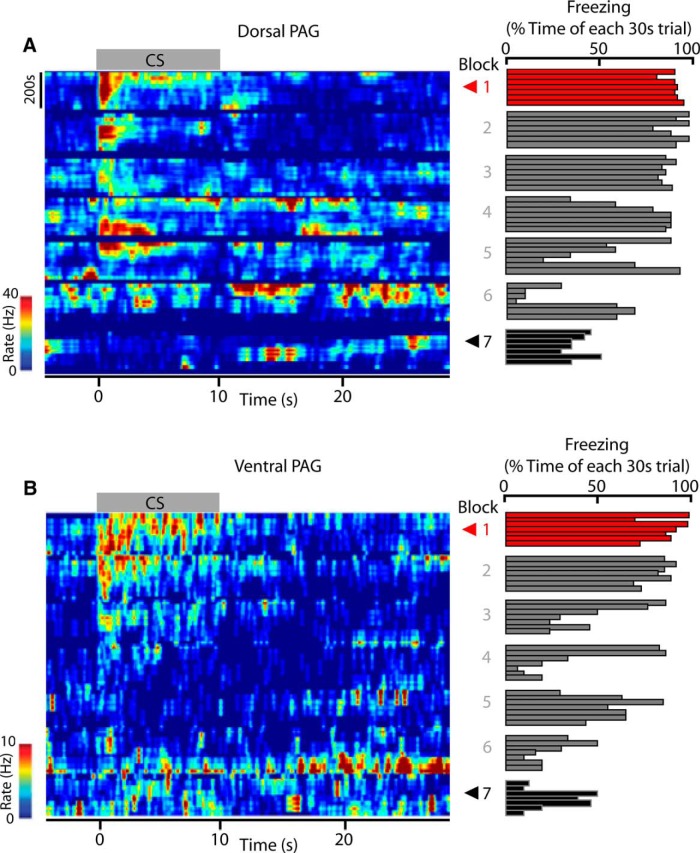
Changes in PAG firing patterns during extinction training. Example heat maps displaying changes in dorsal (***A***) and ventral (***B***) PAG unit firing frequency as a peristimulus plot throughout the complete sequence of extinction training (from CS block 1 to 7). Hotter colors indicate higher firing rates. Red and black arrowheads indicate early (CS block 1) and late (CS block 7) extinction blocks, respectively. Bin size = 100 ms. Time 0 indicates onset of CS and gray horizontal bar at top of each heat map shows CS duration. Percentage time spent freezing during each trial (seven trials per block) is shown to the right of the heat map.

The heat maps of Figure 6, *A* and *B*, illustrate the change in firing pattern of two example units recorded from dPAG and vPAG, respectively, during the full sequence of extinction training, together with the time spent freezing within each trial (right bar charts). Consistent with the data as a whole (Fig. 5), during EE (CS block 1), both units displayed a clear change (in this case an increase) in firing rate during presentation of the CS (Fig. 6, red arrowheads), but, during LE (CS block 7), the same units showed little or no change in activity during presentation of the CS (Fig. 6, black arrowheads).

**Figure 7.**
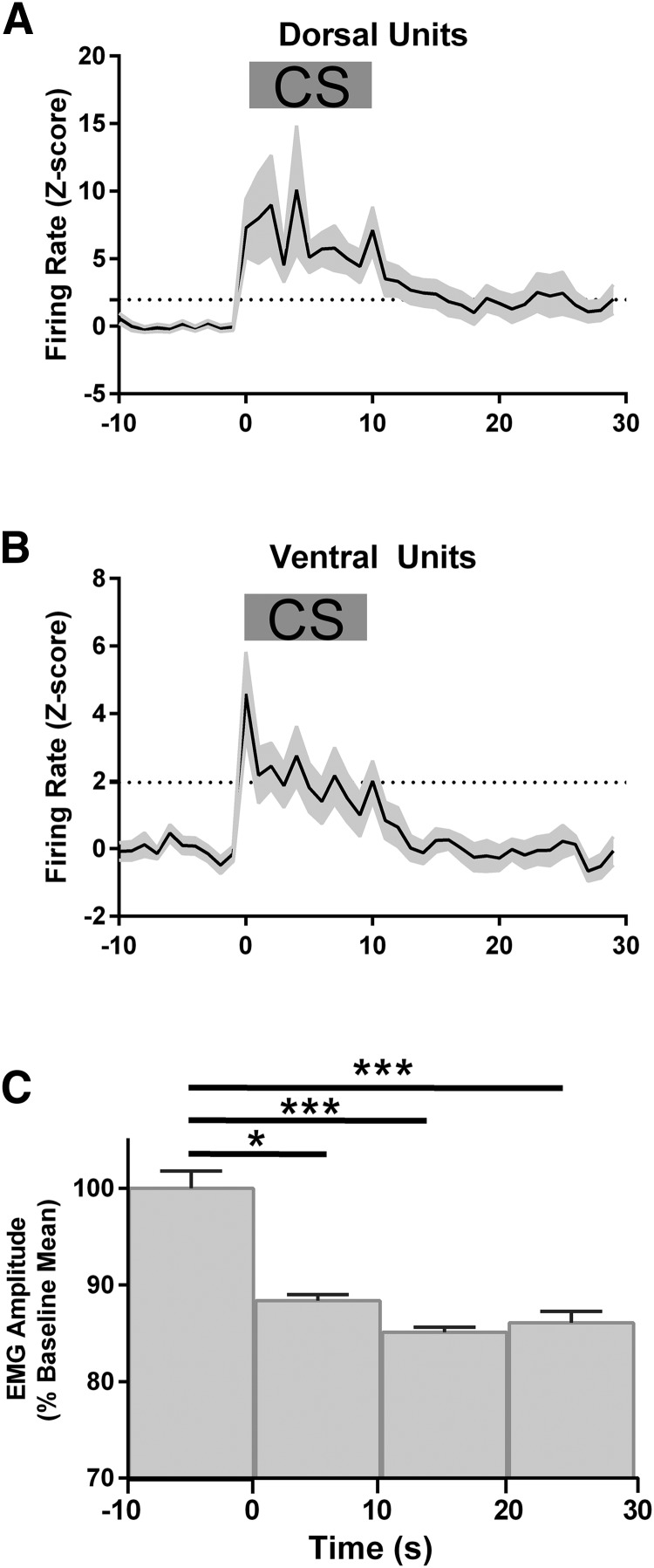
PAG unit and EMG activity during EE. ***A***, PSTH constructed from a sample of 20 dPAG units (*n* = 9 animals) in which neck EMG was simultaneously recorded with unit activity. ***B***, Same as ***A*** but PSTH constructed from a sample of 20 vPAG neurons (*n* = 9 animals). For ***A*** and ***B***, bin size = 1 s; horizontal dotted line indicates *p* = 0.05. ***C***, Histogram of neck EMG amplitude in 10 s bins. Analysis in ***A***–***C*** was based on response to first presentation of CS in EE. In all plots, time 0 indicates CS onset and gray horizontal bar in ***A*** and ***B*** shows CS duration. ***p < 0.001, *p < 0.05, Kruskal–Wallis test with Dunn's *post hoc*, *n* = 9 rats.

Visual inspection of the individual examples illustrated in Figure 6 suggests that changes in cell firing during extinction training are not tightly coupled to the concomitant changes in freezing behavior. For example, when the dorsal unit shown in Figure 6*A* showed an increase in firing in extinction block 1, there was also a high level of freezing behavior, but by extinction training block 3, the same unit displayed a smaller increase in firing while the animal still displayed a high level of freezing behavior. Such findings have implications for the role of PAG in driving motor output. Therefore, to further investigate the apparently weak coupling between PAG activity and behavior, we constructed CS-triggered PSTHs and CS-triggered EMG plots for a sample of dPAG and vPAG units, all of which showed a significant increase in activity (type 1 units) during presentation of the CS in EE (triggered by the first CS; Fig. 7). Significant increases in unit firing in both dPAG and vPAG were time locked to the duration of the CS (falling below significance levels by CS offset; Fig. 7*A*,*B*). In contrast, neck EMG amplitude (which is a reliable marker of freezing; Steenland and Zhuo, 2009) remained significantly reduced after the CS had ended, consistent with a sustained period of freezing that outlasts the CS (*p < 0.05, mean rank difference = 13.3 for −10 to 0 s bin vs 0 to +10 s bin; ***p < 0.001, mean rank difference = 25.0 for −10 to 0 s bin vs 10 to +20 s bin; ***p < 0.001, mean rank difference = 21.3 for −10 to 0 s bin vs 20 to +30 s bin, Kruskal–Wallis test with Dunn's *post hoc*; *n* = 9 rats; Fig. 7*C*).

**Figure 8.**
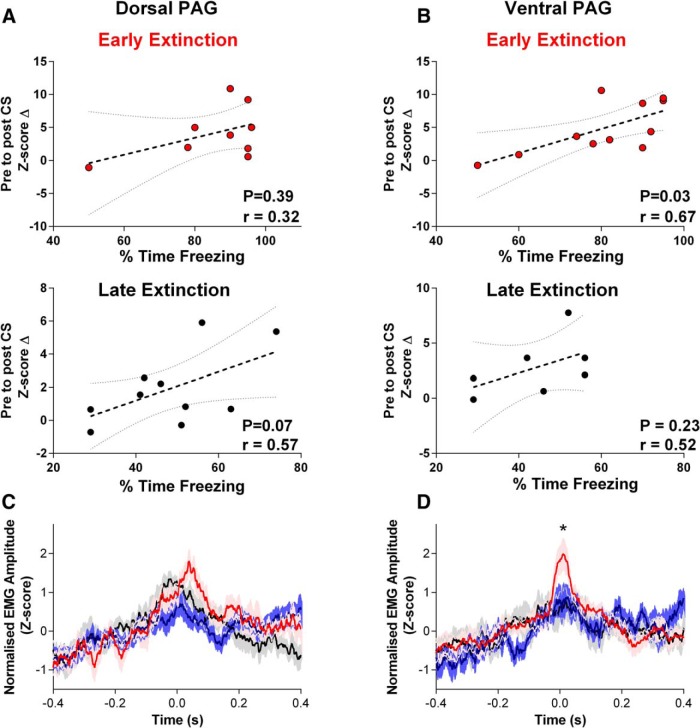
Relationship between PAG and EMG activity. ***A***, ***B***, Spearman correlation between the average CS-evoked changes in firing rate for type 1 dPAG and vPAG units as a function of freezing levels. Sample of dPAG units obtained from *n* = 9 rats during EE and *n* = 10 rats during LE. Sample of vPAG units obtained from *n* = 11 rats during EE and *n* = 7 rats during LE. *z*-score change was computed for each cell and averaged per rat before correlation against freezing level (dashed line). Dotted gray lines indicate 95% confidence interval. ***C***, Spike-triggered EMG average using spiking activity of 20 dorsally located PAG units recorded from 9 rats during 10 s sample of immobile behavior preconditioning (black line), during the 0–10 s period of CS delivery in EE (red line), and 10–20 s post CS (blue line). EMG data were rectified, smoothed (0.025 s), and expressed as a *z*-score. Shading indicates SEM. ***D***, Same as ***C*** but constructed using spikes from ventrally located units (*n* = 20 units, 9 rats). *p < 0.05, ANOVA with Tukey's *post hoc* test between peak in EE spike-triggered average (red) versus baseline (black).

The data in Figure 7 therefore indicate, at a population level, that changes in EMG activity induced by the CS during EE outlast the associated increases in firing rate of both dPAG and vPAG units. However, group analysis may obscure effects at the level of single units. Figure 8, *A* and *B*, therefore plot for individual type 1 units, the change in normalized firing rate between preconditioning and postconditioning, as a function of freezing for dPAG and vPAG units, respectively. For type 1 units located in dPAG, we found no significant correlation between change in firing rate and the extent of freezing in either EE or LE (Spearman correlation *p* = 0.39, *r* = 0.32 during EE, bottom, *p* = 0.07, *r* = 0.57 during LE; Fig. 8*A*, top). However, for type 1 units recorded from vPAG, we found a significant positive correlation between change in firing rate and freezing level during EE (Spearman correlation *p* = 0.03, *r* = 0.62; Fig. 8*B*, top), but not during LE (Spearman correlation *p* = 0.23, *r* = 0.52; Fig. 8*B*, bottom).

To further investigate the relationship between PAG activity and behavior, we constructed spike-triggered averages of neck EMG. Figure 8, *C* and *D*, shows the averaged data, respectively, for dorsal units (*n* = 20) and ventral units (*n* = 20). The plots show the results: (1) when rats were displaying immobility before conditioning (baseline, black plots); (2) during EE and the 10 s period in which the CS was presented, when conditioned freezing starts to occur (red plots); and (3) during EE and the 10–20 s period after the CS when conditioned freezing continues to occur (cf. Fig. 8*C*, blue plots). For dorsal units, the spike-triggered EMG was similar for the baseline sample and during both CS time periods in EE training. The small peak during EE, ∼45 ms after the onset of the CS, was not significantly different compared with peaks during baseline or post-CS [baseline *z*-score 0.97 ± 0.3 versus EE peak *z*-score 1.8 ± 0.3; ANOVA with Tukey's *post hoc* tests between peak in EE spike-triggered average (red) vs baseline (black) or post CS (blue), *p* = 0.07, *F*_(2,24)_ = 2.9, *n* = 9 rats; Fig. 8*C*].

In contrast, for ventral units, there was a statistically significant peak (Fig. 8*D*, asterisk) in the spike-triggered EMG compared with baseline during the 10 s time period of the CS in EE, but not during the 10 s time period immediately after the CS [baseline *z*-score 0.68 ± 0.4, EE peak *z*-score 2.05 ± 0.4; ANOVA with Tukey's *post hoc* tests between peak in EE spike-triggered average (red) vs baseline (black), *p* = 0.02, *F*_(2,24)_ = 4.4, *n* = 9 rats; Fig. 8*D*]. The significant peak in cross-correlation occurred ∼10 ms after the onset of the CS.

## Discussion

The key findings from the present study are as follows: (1) in response to an innate fear stimulus, dPAG units show more robust increases in their activity than vPAG units; (2) the pattern of response of dPAG and vPAG units during extinction of a fear-conditioned response can be divided into four types: type 1 (increase), type 2 (no change), type 3 (biphasic), and type 4 (decrease); (iii) for dPAG and vPAG units, the proportion of the different types of response is similar during EE (mainly type 1) but differs during LE, when, (a) despite there being a general increase of type 2 and 4 cells, the majority of dPAG cells remain as type 1 and (b) type 1 vPAG units in EE continue to respond robustly in LE (i.e., a subpopulation of both dPAG and vPAG units are extinction resistant); and (4) the activity of vPAG units, but not dPAG units, is cross-correlated with EMG during the initial stage of conditioned freezing.

### Comparison with previous studies

Most PAG studies to date have involved behavioral observations after localized lesions or stimulation or charting regions of cellular activation with FOS (Bandler and Depaulis, 1988; Carrive et al., 1997; Vianna et al., 2001b; Bittencourt et al., 2005). A general consensus has emerged that dPAG directs motor outputs to innate fear, including flight (Carrive, 1993; Bandler and Shipley, 1994; Vianna et al., 2001b; Kim et al., 2013; Halladay and Blair, 2015; Wang et al., 2015), whereas vPAG is required for the expression of fear-conditioned freezing; that is, its activation leads to a general cessation in movements and a fixed motionless posture (LeDoux et al., 1988; Kim et al., 1993; De Oca et al., 1998). Conditioned fear is related to a marked increase in FOS expression in vPAG (Carrive et al., 1997). Therefore, the dorsal and ventral columns of PAG are thought to drive mainly opposing types of behavior.

Our finding that dPAG cells fire more robustly than vPAG cells to an innate fear stimulus of predator odor is consistent with this functional distinction. It should however be noted that the cat odor was always presented after the foot-shock conditioning and extinction training, which raises the possibility that the innate responses may have been modified by this previous experience. Further studies would be required to explore any interaction between innate and conditioned fear on neural activity in the PAG. In relation to fear conditioning, we found that, during EE (when retrieval of the conditioned freezing response is maximal), both dPAG and vPAG neurons display similar patterns of response. In relation to vPAG, our findings show that neural activity in this region of PAG is time locked to a CS that elicits freezing behavior, particularly the onset of the conditioned tone. Importantly, vPAG cells did not respond to the same auditory tone when it was unconditioned. This provides evidence that vPAG activity is related to the associative conditioning rather than the sensory stimulus. Moreover, because vPAG cells displayed significant coupling to conditioned EMG activity during EE (with spikes preceding the peak in EMG activity by ∼10 ms), this is consistent with vPAG cells driving the initial motor response, although it should be emphasized that this does not exclude the possibility that such cells might also encode fear memory.

Any significant increases in vPAG activity were short lasting (typically 1–2 s in duration) and did not match the longer-lasting EMG activity associated with the conditioned freezing response (which in EE lasted at least 18 s). Based on the sample of cells that we recorded, the pattern of neural activity observed in vPAG is therefore inconsistent with maintaining freezing behavior. This is contrary to the model proposed by Burgos-Robles et al. (2009), which predicts that PAG neurons would show sustained conditioned responses. Instead, our data suggest vPAG cells could drive initial aspects of the conditioned motor response, but other CNS structures may be involved in sustaining the behavior. Further studies would be required to explore this possibility, for example, by studying the effect of delivering brief stimulus pulses to vPAG.

One candidate structure for sustaining the conditioned response is the prelimbic cortex (Blum et al., 2006; Sierra-Mercado et al., 2011). Prelimbic cells can show tonic increases in firing activity that mirror the time course of freezing to a conditioned tone (Burgos-Robles et al., 2009). However, prelimbic activity starts 100 ms after the onset of a conditioned tone, whereas changes in PAG activity occur sooner (∼30–35 ms in the current study; ∼30 ms in Halladay and Blair, 2015). By comparison, responses in lateral amygdala occur ∼15 ms after a conditioned tone (Quirk et al., 1997), suggesting that both vPAG and prelimbic activity are initiated by the amygdala. However, in rats, the prefrontal cortex is also the major recipient of corticopetal projections arising from the PAG (Herrero et al., 1991), raising the additional possibility that vPAG contributes directly to driving the conditioned responses in prelimbic cortex.

Our finding that dPAG neurons display a similar pattern of response as vPAG cells to the CS during EE is surprising. Carrive et al. (1997) found less FOS expression in this region of PAG as a result of conditioned fear and lesions of dPAG do not block the expression of conditioned fear behavior (Leman et al., 2003). Also, Halladay and Blair (2015) found that dPAG neurons were responsive to a CS that led to movement excitation (i.e., increased movement, including flight), but not inhibition (i.e., suppression of movement, including freezing). However, one important difference from our study is that, rather than extinction learning, Halladay and Blair (2015) examined retrieval of conditioned responses in well trained animals that received daily reinforcement of the fear-conditioning paradigm. Therefore, their animals may have been in a more aroused/fearful state with a greater expectancy of the unconditioned stimulus, which may influence the pattern of PAG activity.

However, consistent with our findings, approximately half of their dPAG cells increased activity during the initial part of the auditory CS regardless of whether the conditioned behavioral response was excitatory or inhibitory. Chemical or optogenetic activation of dPAG can induce both flight and freezing (Krieger and Graeff, 1985; Deng et al., 2016). Our finding that dPAG cell responses are time locked to a CS associated with freezing is therefore consistent with the possibility that they may be related to fear memory and/or freezing behavior. Like vPAG cells, dPAG cells did not respond to the same auditory tone when it was unconditioned; however, unlike vPAG cells, they did not display a significant coupling to conditioned EMG activity during EE.

Together, these results therefore suggest that dPAG activity during EE may be more related to the recall of fear memory than sensory or motor aspects of the behavioral response. Consistent with this is the finding that lesions of dPAG can enhance both the acquisition and expression of conditioned fear responses, suggesting an inhibitory role in learning and memory (De Oca et al., 1998).

### Types of response

Studies of neural activity in central circuits associated with fear have focused on the amygdala (Muramoto et al., 1993; Quirk et al., 1995). Because the direct amygdalo–PAG pathway is thought to be inhibitory, it has been predicted that target neurons in PAG will reduce their activity during fear-conditioned behavior (Duvarci et al., 2011). However, our results indicate that most PAG cells increase their activity during fear retrieval, which implies that the primary influence of amygdala on the PAG may be on inhibitory interneurons, leading presumably to disinhibition of other PAG cells. A recent optogenetic study in mice provides direct evidence to support this interpretation (Tovote et al., 2016).

### Extinction susceptible and resistant cells

During LE, there was an increased incidence of type 2 and type 4 cells in both dPAG and vPAG; that is, an increased proportion of cells that displayed either no change or a reduction in response to the unreinforced CS. Such activity is consistent with neural plasticity associated with extinction and is strikingly similar to the pattern described previously for the amygdala (Hobin et al., 2003; Herry et al., 2008; An et al., 2012). A substantial population of dPAG and vPAG cells (almost half) also responded during LE. In particular, type 1 cells in vPAG displayed an increase in firing rate that was not significantly different compared with those responses observed during EE (i.e., were extinction resistant). According to general learning theory, extinction is not the erasure of the associative memory, but rather is a form of context-dependent inhibitory learning that temporarily suppresses expression of the conditioned response (Todd et al., 2014). The extinction resistant cells reported here may therefore contribute to the persistence of fear memory after extinction, analogous to the pattern of activity in the amgydala and prefrontal cortex (Burgos-Robles et al., 2009; An et al., 2012).

Our results therefore suggest that PAG is not, as widely considered, merely the final executor for top-down drive of fear-related motor output, but may also be concerned with maintaining the memory trace of conditioned fear.
